# Universally Distributed Single-Copy Genes Indicate a Constant Rate of Horizontal Transfer

**DOI:** 10.1371/journal.pone.0022099

**Published:** 2011-08-05

**Authors:** Christopher J. Creevey, Tobias Doerks, David A. Fitzpatrick, Jeroen Raes, Peer Bork

**Affiliations:** 1 Animal and Bioscience Research Department, Teagasc, Grange, Dunsany, Ireland; 2 European Molecular Biology Laboratory, Heidelberg, Germany; 3 Department of Biology, National University of Ireland Maynooth, Maynooth, Ireland; 4 VIB Department of Molecular and Cellular Interactions, Vrije Universiteit Brussels, Brussels, Belgium; University of Wyoming, United States of America

## Abstract

Single copy genes, universally distributed across the three domains of life and encoding mostly ancient parts of the translation machinery, are thought to be only rarely subjected to horizontal gene transfer (HGT). Indeed it has been proposed to have occurred in only a few genes and implies a rare, probably not advantageous event in which an ortholog displaces the original gene and has to function in a foreign context (orthologous gene displacement, OGD). Here, we have utilised an automatic method to identify HGT based on a conservative statistical approach capable of robustly assigning both donors and acceptors. Applied to 40 universally single copy genes we found that as many as 68 HGTs (implying OGDs) have occurred in these genes with a rate of 1.7 per family since the last universal common ancestor (LUCA). We examined a number of factors that have been claimed to be fundamental to HGT in general and tested their validity in the subset of universally distributed single copy genes. We found that differing functional constraints impact rates of OGD and the more evolutionarily distant the donor and acceptor, the less likely an OGD is to occur. Furthermore, species with larger genomes are more likely to be subjected to OGD. Most importantly, regardless of the trends above, the number of OGDs increases linearly with time, indicating a neutral, constant rate. This suggests that levels of HGT above this rate may be indicative of positively selected transfers that may allow niche adaptation or bestow other benefits to the recipient organism.

## Introduction

From the earliest comparative genomic studies it was obvious that horizontal gene transfer (HGT) occurred frequently [1]–[3] and would impinge upon our efforts to understand the evolutionary history of all life [4]–[8]. HGT has been shown to occur between both closely and distantly related organisms, in both fast and slowly evolving gene families [9]–[12]. While proteins with multiple interactions are not immune to transfer [13], [14], they seem to undergo fewer HGT events providing evidence for the “complexity hypothesis” [1], [15]. Efforts have been made to quantify the effect of HGT in completely sequenced organisms [7], including estimating the rates of HGT across all organisms [16], [17]. Although barriers for HGT have been revealed [15], [18]–[20] fundamental factors that influence the rate of HGT remain to be identified, proven and quantified [21].

The vast majority of gene families shared by multiple organisms have undergone horizontal transfer events at some point in their evolutionary history [22]. However, as the majority of these families are present in multiple copies in at least some organisms, factors influencing the correct identification of orthologs, or the occurrence of duplication and multiple loss events [23], [24] can mar the identification of fundamental factors influencing the rate of HGT in these genes. In order to address this, selection of gene families for analysis must minimise the potential for inclusion of these kinds of events. To that end, we focussed on the approximately 1% [25] of gene families that are universally single copy [14], and likely have been functionally preserved since the emergence of the three domains of life. When these genes are successfully subjected to HGT, they should maintain the interactions of the original copy that they displace (orthologous gene displacement (OGD) [26]). This should be an extremely rare process as two copies of genes that are part of large multi subunit complexes (like the genes studies here) will not be tolerated due to dosage effects [20]. Furthermore, it has been suggested that even marginal differences in sequence identity between the displaced copy and it's replacement is enough to cause a marked decrease in fitness of the acceptor organism [27], requiring compensatory evolutionary change to occur. This suggests that the successful fixation of an OGD in these genes requires overcoming the most stringent barriers of any horizontal transfer event [20]. These characteristics make HGTs in these genes potentially important for the elucidation of constraints and promoting factors of HGT in general.

Here we introduce an automated approach of detecting OGD events in universal single gene families that i) is based on a statistical framework, ii) has the ability to detect ancient events and iii) can determine not only the recipient but also the donor organism. Applied to 40 universal single-copy genes in 191 species with completely sequenced genomes, we explored parameter-space to identify optimum settings. The surprisingly large number of robust orthologous gene displacements identified allowed us to quantify factors that have governed the occurrence of horizontally transferred orthologous gene replacements since the last universal common ancestor (LUCA).

## Results and Discussion

### Detection of OGDs

Our approach of automatically detecting OGDs is based on the comparison of the phylogenetic signal of each individual gene family (gene tree) to the combined phylogenetic signal of all the genes used in the study (combined tree). The theory behind this approach is that phylogenetic signal is cumulative as opposed to homoplastic noise which is dispersive [28], therefore strong disagreement between the combined phylogenetic signal of all the gene families and that of any one gene may be representative of a homoplastic event like HGT [29]. Our method depends on the genes studied sharing a core phylogenetic history, such as was demonstrated previously for the informational genes used in this analysis [14]. This is a similar idea to the commonly used approach of comparing a species tree with a gene tree to identify HGT, but allows the identification of HGT in sets of functionally related genes with a shared core phylogenetic history (like information processing genes, or genes in operons) in organisms where a species tree concept may not apply or be difficult to reconstruct (like in prokaryotes).

**Figure 1:**
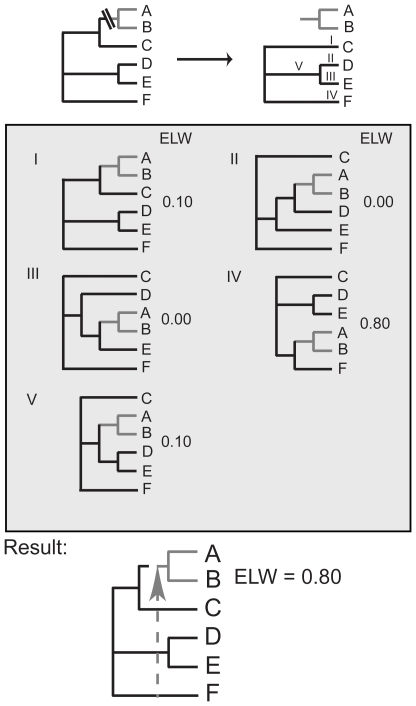
The assessment of possible OGDs on the tree. The calculation of OGDs involves the assessment of the likelihood of the placement of every branch of the unrooted tree at every possible position for each of the gene families separately. 1) The branch leading to Species A and B is to be assessed and is pruned. It is then replaced at every possible position on the remaining tree (I to V above) and the likelihood of each is assessed. 2) When all likelihoods have been assessed the expected likelihood weight (ELW) of each is calculated. If any placement except the original position receives an ELW of at least 0.65 (implying high support) this is considered a putative transfer event (as in IV above). The branch with the best placement is considered the donor and the recipient is the branch that was assessed at each position (A and B above). 3) To be considered an OGD the path-length distance of the original position of the clade to its best placement is calculated. If this distance is greater than 0.4 (thereby avoiding the possibility of wrongly identifying phylogenetic uncertainty as an OGD), the placement is considered a putative OGD.

We identified 40 gene families that are universally distributed in single copy across all life and used their combined phylogenetic signal to construct a tree. This tree was then used in an exhaustive maximum likelihood procedure where the sequence data for each individual gene family was used to determine the best phylogenetic placement of every branch of the tree (inspired by [30] where the concept had been used to infer species compositions from metagenomics samples), identifying when this indicated a possible orthologous gene displacement (Figure 1).

**Figure 2:**
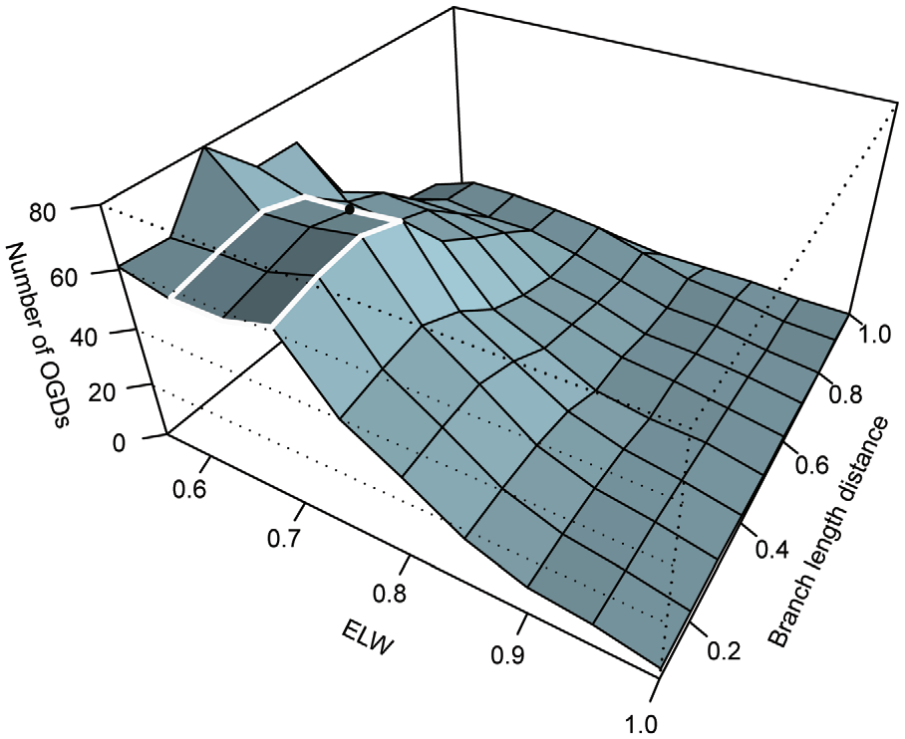
Parameter exploration. The surface illustrates the effect on the number of OGDs detected of all combinations of ELW cut-offs from 0.55 to 1.0 (in steps of 0.5) with all combinations of branch length distance cut-offs from 0.1 to 1.0 (in steps of 0.1). Highlighted in white around its edges is the range of parameters that converge on the same level of HGT detected. The black dot indicates the settings chosen for the purposes of investigating the underlying factors influencing orthologous gene displacement in these genes.

**Figure 3:**
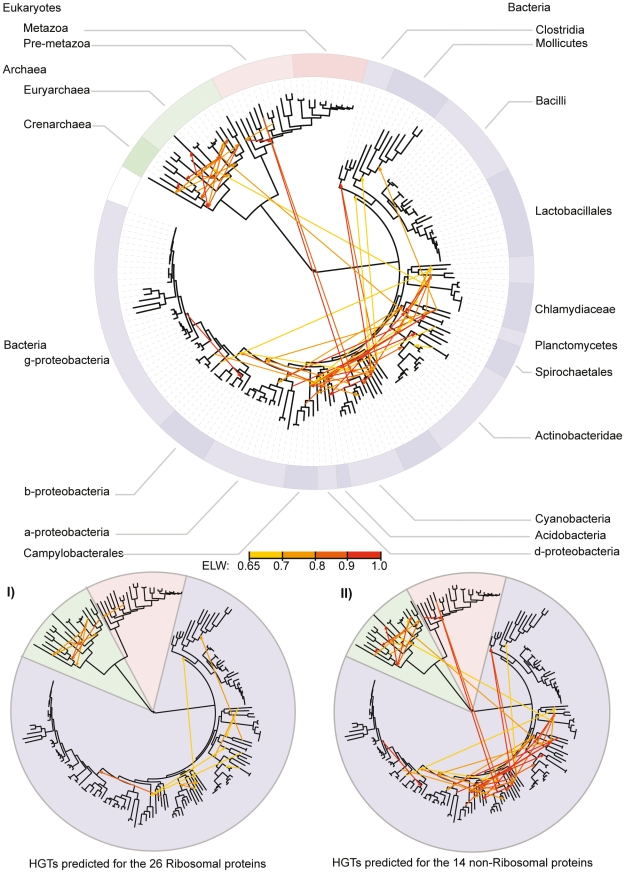
Predicted OGDs. In the main figure the total predicted OGDs for all 40 gene families (as visualised using the iTOL web server [45]) project onto the tree constructed using the combined phylogenetic information from all 40 gene families. The colours of the arrows represent the statistical certainty of the transfer, from yellow (0.65) to red (1.00). The Eukaryotes are marked in reds, the Archaea are in greens and the Bacteria in Blues. The minor figures represent I) the total OGDs predicted for all the Ribosomal proteins (26 gene families) and II) the total OGDs predicted for all the non-Ribosomal proteins (14 gene families).

To identify the “best” parameters for our analysis and to examine the robustness of results to different parameter selection, a wide range of parameters were explored for the number of OGDs identified (Figure 2). Depending on the settings used the number of HGTs detected ranged from 0 to 80, however interestingly the parameter exploration identified a range of settings where the number of OGDs plateaued at around 65 (Figure 2). Other parameter settings existed that increased the number of OGDs detected above that observed in the plateau in Figure 2 and may represent false positives. For instance we used the expected likelihood weight (ELW) to test the support for an alternative position for a branch (and hence a possible OGD event). In likelihood weighting each sampled tree is weighted by the likelihood that it accords with the evidence (the alignment). Trees for which the alignment is unlikely are given less weight. The sum of the weights calculated for all the trees tested equals 1. This allows the quantification of the weight of evidence supporting each of the un-rejected trees and their ranking according to their support of the evidence in the alignment [31] (see the methods for more detail). Using an ELW cut-off of 0.55 and minimum path length distance of 0.3, increased the number of OGDs observed to 80 (Figure 2). However we feel that the parameters at the plateau at around 65 OGDs represent an optimum, given the shape and branch-lengths of the combined tree (Figure 3). To further minimise the possibility of false positives we chose one of the most conservative of the settings in the plateau to identify putative HGTs for further analysis: an expected likelihood weight (ELW) [31] of 0.65 (representing a single un-rejected tree that contributes 65% of the total weighted evidence in support of the alignment from all the un-rejected trees – see methods for more details) and a minimum the path length distance across the tree from the best placement of the branch to its placement on the combined tree of 0.40 substitutions per site, [32] (Figure 2).

Using these settings, 68 orthologous gene displacement events were identified in the 40 gene families analysed (Figure 3) consisting of 38 OGDs from the subset of 31 gene families previously processed using a manual approach [14] (including, all 7 OGDs found in that analysis) and a further 30 OGDs from the additional 9 gene families analysed here (Table 1).

**Table 1:** The number of OGDs predicted for each of the 40 universal single-copy gene families used in this study.

OGDs	Interactions	Gene family ID	Description
0	111	COG0048	Ribosomal protein S12
0	146	COG0052	Ribosomal protein S2
0	115	COG0080	Ribosomal protein L11
**0**	**158**	**COG0085**	**DNA-directed RNA polymerase**
0	119	COG0087	Ribosomal protein L3
0	111	COG0091	Ribosomal protein L22
0	106	COG0092	Ribosomal protein S3
0	108	COG0093	Ribosomal protein L14
0	121	COG0094	Ribosomal protein L5
0	119	COG0096	Ribosomal protein S8
0	110	COG0097	Ribosomal protein L6P/L9E
0	126	COG0100	Ribosomal protein S11
0	123	COG0184	Ribosomal protein S15P/S13E
0	114	COG0200	Ribosomal protein L15
0	80	COG0201	Preprotein translocase subunit SecY
**0**	**9**	**COG0552**	**Signal recognition particle GTPase**
**1**	**122**	**COG0088**	**Ribosomal protein L4**
1	124	COG0098	Ribosomal protein S5
1	118	COG0099	Ribosomal protein S13
1	114	COG0103	Ribosomal protein S9
**1**	**111**	**COG0185**	**Ribosomal protein S19**
1	116	COG0186	Ribosomal protein S17
1	122	COG0197	Ribosomal protein L16/L10E
1	147	COG0522	Ribosomal protein S4
2	115	COG0081	Ribosomal protein L1
2	111	COG0102	Ribosomal protein L13
2	24	COG0172	Seryl-tRNA synthetase
**2**	**11**	**COG0215**	**Cysteinyl-tRNA synthetase**
2	111	COG0256	Ribosomal protein L18
2	25	COG0495	Leucyl-tRNA synthetase
**2**	**19**	**COG0541**	**Signal recognition particle GTPase**
3	12	COG0016	Phenylalanyl-tRNA synthetase alpha subunit
3	128	COG0049	Ribosomal protein S7
**3**	**129**	**COG0090**	**Ribosomal protein L2**
3	153	COG0202	DNA-directed RNA polymerase
3	8	COG0533	Metal-dependent protease
4	31	COG0525	Valyl-tRNA synthetase
6	22	COG0012	GTP Binding Protein
**10**	**17**	**COG0018**	**Arginyl-tRNA synthetase**
**11**	**42**	**COG0124**	**Histidyl-tRNA synthetase**

**Figure 4:**
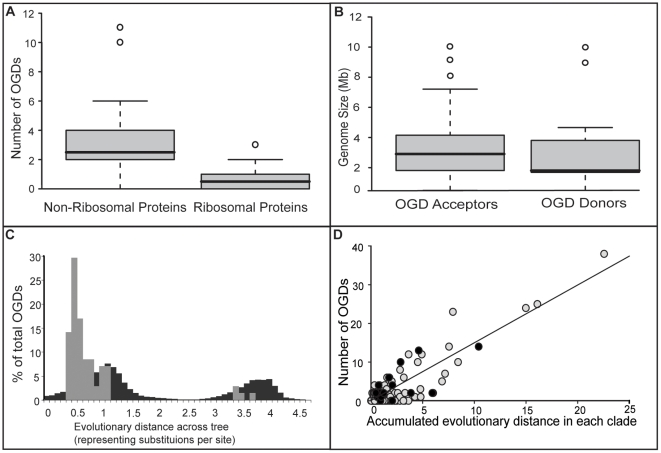
Factors influencing OGDs in single copy genes. A) There is a significant correlation with the biological function of the proteins and their propensity to OGDs (p<0.01 Wilcoxon Mann-Witney test). B) The genome size of the acceptors are significantly larger than the donors (p = 0.02 Wilcoxon Mann-Witney test). C) The OGDs appear to occur between more closely related organisms than would be expected from the shape and structure of the tree in Figure 3 (p = 0.001, Kolmogorov-Smirnov test). In black: all 103,210 possible distances between donors and acceptors based on the tree in figure 3. In grey: the distribution of evolutionary distances between the observed donors and acceptors. The bimodal nature of the distributions stems from the difference in phylogenetic distances between taxa when they are in the same Domain of life versus when they are from different Domains. See Figure 3 for an illustration of the phylogenetic distances involved. D) The rate of OGDs is significantly correlated with evolutionary time/opportunity calculated for all 380 branches of the tree (in grey) and for the 15 chosen independent groups of organisms (in black) p = 0.005.

The gene families in bold are the 9 gene families analysed here in addition to the 31 previously analysed using a manual approach [14]. “Interactions” are the number of interactions with other gene families predicted for this gene family using the String 7 database [33] with a cut-off of 0.7. A significant negative correlation was found to exist between the number of Interactions predicted and the number of OGDs detected for each of the genes (see Figure S3). There was also a significant correlation between the number of OGDs detected and whether the gene coded for a ribosomal protein or not (Figure 4a).

The rate of orthologous gene displacement should represent the lower bound of HGT in all gene families as transfers of informational genes should be already rare and displacement represents yet another barrier. Therefore, it came as a surprise that even with the limited species set used here we found up to 50% of the ribosomal proteins analysed had undergone OGD according to our stringent method (Table 1). While in the ribosomal genes a rate of 0.76 OGDs per family in the gene's life span is observed across the 191 species examined (Table 1), proteins with fewer predicted interactions [33] (e.g. tRNA synthethases and GTPases) have a 4-fold higher rate of 3.4 OGDs per gene family (Figure 4a) across the same set of 191 species. For all 40 informational genes from 191 species the average rate is 1.7 OGDs per gene family, which is similar to recent estimates of a lower bound of 1.1 HGT per gene family and gene family life span [16].

### Factors associated with HGT

Given the considerable amount of OGD discovered, 5 different possible factors that may promote or inhibit the rate of horizontal transfer in these universally distributed single-copy genes were investigated.

#### 1) GC content

Using the GC content of the 191 extant species we estimated the GC content of predicted ancestors at each of the internal branches of the tree in Figure 3. When we compared the GC content of the donors and acceptors however we found no correlation between the GC content of the donor and acceptors of the OGDs detected. Even though having a similar GC content between the donor and acceptor has been suggested to influence the successful fixation of HGTs [34] our results suggest that it doesn't influence the rate of OGD (Figure S1).

#### 2) Habitat

Using habitat information from cultivated strains in culture collections we estimated the habitat specificity (how specialised the organism's niche was) of predicted ancestors at the internal branches of the tree in Figure 1 (see methods). We then examined the habitat specificity of the donor and acceptor of each OGD detected. We found that the habitats were not more similar than would be expected by chance (Figure S2); however this result may be undermined by the amount of time since the OGD events occurred. In this timeframe, it is entirely likely that the present-day descendents of the donors or acceptors have different habitats to their ancestors.

#### 3) Genome size

Using the genomes of the extant 191 species, the genome sizes were estimated for the predicted ancestors at the internal branches of the tree in Figure 3 (see methods). Comparing the genome size of the species involved in the OGDs detected we found that the genomes of the acceptors were significantly bigger than the donors (p = 0.02 using wilcoxon rank sum test with continuity correction), indicating that species with larger genomes are more likely to undergo OGD (Figure 4b).

#### 4) Biological Function

For the 40 genes analysed we found a significant negative correlation between the number of predicted interactions in which a gene was involved and the number of OGDs found (Table 1 and Figure S3). Furthermore we found that ribosomal proteins underwent significantly fewer OGDs than genes from other functional categories (Figure 4a) supporting biological function as a barrier to successful displacement (i.e. the complexity hypothesis [1]). However, on a more fine-grained level there was no difference within the ribosomal proteins based on their number of interactions, assembly order, or whether they bound to the large or small subunit (Figure S4). Only anecdotal evidence supported any discrimination, that of whether the proteins bind directly to the RNA core or not (Figure S4).

#### 5) Evolutionary distance

The evolutionary distance since the last common ancestor (LCA) of the donor and acceptor was calculated for each of the OGDs detected (see methods). These distances were compared to distribution of distances between all possible donor and acceptors given the process of evolution described in the tree in Figure 3. We found that the distribution of distances for the OGDs detected were significantly shorter than would be expected from the shape of the tree (p = 0.001 using the Kolmogorov-Smirnov test) confirming [27] that evolutionary divergence influences the success of orthologous gene displacements (Figure 4c).

Interestingly of the 5 factors tested, the two that were found to have no effect (GC content and Habitat) may have been affected by the timescales involved in this analysis, but there is also the possibility that our dataset was not large enough to identify a clear signal. However we did find 3 positive factors even with such impediments, so we further investigated the properties of the OGD signal in the dataset.

### Dating

Even though we could find no signal for similar habitats between the donors and acceptors of the OGDs the existence of the transfer event confirms their presence in the same place at the same time (or at least in the presence of an intermediary) allowing different parts of the phylogeny to be dated to the same time-point. This dating is needed to understand the impact of transfers in the different domains of life, phyla or clades. Indeed, when the distance from the donor and acceptor to their last common ancestor was calculated (and using the branch lengths of the donor or acceptor branch as confidence intervals), 53 of the 68 HGTs overlapped as would be expected if the branch lengths involved are a good representation of time (Table S1) and can be used as basis to derive rates of OGD. This is illustrated in figure S5, where the most highly supported horizontal transfers fit horizontally onto the tree (except for two minor clades) (see Figure S5).

### Rate of OGD

We thus counted the number of OGDs that had been accepted within different time frames designated by each of the clades of the tree in figure 3. The total evolutionary branchlength within a clade was plotted against the number of OGDs sucessfully fixed into the clade. As nested clades are not independent of each other we chose independent examplar clades for the purposes of calculating robust statistics (see methods). We found a strong fit of the data to a linear model (p = 0.005) (Figure S6) between these independent clades and evolutionary distance/time (marked in black in Figure 4d) and found that the data was linear when tested using both a linearity test (p<0.005) (Figure S7) and using a runs test (p>0.5) (Figure S8), indicating a constant rate of OGD in these universal single-copy genes.

We further tested if the linear relationship was as a result of the cutoffs used during the identification of the OGDs by recalculating the rates of OGD identified using the results obtained during the parameter exploration. We found that changing the cutoffs had little effect on the resulting linear relationship (see Figure S6).

### Phylogenetic classification

Supporting the hypothesis that OGDs occur randomly at a constant rate, we found that the number of OGDs occurring in the Archaea and Bacteria was consistent with the amount of evolution in each Domain, even though Bacteria possess mechanisms of gene uptake absent from the Archaea [21], [35]. This is not to say however that some clades do not accumulate more transfers than others, indeed the Bacilli clade was found to have no OGDs compared to the Spirochaete clade which contained 6, despite both clades having a similar amount of phylogenetic diversity (1.96 substitutions per site compared to 1.78 substitutions per site).

Thus, although i) some clades accumulate more OGDs than others, ii) some gene families and iii) genomes are more likely to be subjected to OGD than others and iv) OGD occurs preferably between more closely related genomes, it appears that those events measured over long time periods across all life, occur at a constant rate.

### Conclusion

By developing a new method for the identification and position-specific masking of OGDs, we could quantify the large number of events that have occurred in 40 gene families that have been in existence since the last universal common ancestor. OGDs are likely to represent the type of HGT with the most barriers to overcome in order to be fixed into a population and so the factors involved in their occurrence are probably fundamental to all HGTs. We have studied a number of these factors and even though we used a heuristic approach which is limited by the taxon sampling available and by the deterioration of phylogenetic signal over time, we could still show the influence of some of them., However we found that even though there was heterogeneity in the number of OGDs that occurred in different groups of species and in different genes, when taken as a whole OGDs occur at a constant rate over the lifetime of the gene families analysed. This is similar to the situation in DNA sequences that have rate heterogeneity across sites, but overall show a relatively constant rate of evolution. The constant rate of OGD identified here indicates the existence of a baseline, representing the rate at which neutral HGTs occur and over which selectively advantageous transfers into the acceptor organism may happen. We expect that a slightly modified version of our method could also be applied to gene families with a small number of duplications, allowing the identification of donors and acceptors in a much larger fraction of available genes and the verification of these results in a more general context.

## Materials and Methods

### Data Used

Gene families were selected from the orthologous groups defined in the String database (version 6) [36] based on their being present in single copy in all 191 species sampled (see Table 1 for more details). In some gene families a gene family was predicted to be in single copy in all but one species. In these cases a manual analysis of the sequences was carried out to identify situations where the potential paralog was a misannotation or an inactivated copy of the gene (as identified by multiple stop codons in the sequence). Carrying out this manual analysis allowed the confirmation of a unique ortholog for each organism in these gene families. This analysis identified 40 universal single copy gene families, 31 of which had been previously identified [14].

### Combined tree construction

A phylogeny was constructed of the combined phylogenetic signal of all 40 gene families, following a similar procedure as carried out for the 31 gene families previously identified. [14]. For each of the 40 gene families three separate alignments were created which divided the sequences according to whether they were sequenced from Archaeal, Bacterial or Eukaryotic organisms. These sequences were aligned separately using the default settings in muscle (version 3.6) [37]. Gblocks [38] was then used to curate the alignments by removing ambiguously aligned positions using relaxed settings as follows: allow gap positions  =  with half; minimum length or a block = 2 and default settings for all other options. Then for each gene family, these curated alignments were profile aligned using the default settings in muscle [37], by first profile aligning the eukaryotic and archaeal sequences, and then profile aligning the result with the bacterial sequences. Finally the curated profile alignments of all 40 gene families were concatenated together resulting in an alignment of 13,206 amino acid positions. One-hundred bootstrapped replicates were constructed from this alignment for the purposes of phylogeny reconstruction using seqboot from the Phylip package [39]. For each bootstrapped alignment a phylogeny was constructed using a maximum likelihood procedure as implemented in phyml version 3.0 [40] with the JTT model of evolution (model of nucleotide substitution  =  JTT) and assuming heterogeneous site rates (one category of substitution rate  =  no) as described by a gamma distribution with an estimated alpha (Gamma distribution parameter  =  estimated). The gamma distribution was summarised into 4 site rate categories for the purposes of phylogeny reconstruction (Number of substitution rate categories = 4). The default settings were used for all other options. A phylogeny was also constructed using the same settings for the original un-perturbed alignment. The support values calculated for each internal branch of this tree was taken from the result of the bootstrap analysis using clann (with the “consensus guidetree = yes” command) [41], resulting in a phylogeny that contained both branch lengths and support values (Figure 3). The resulting phylogeny was rooted at the midpoint between the bacteria and the node leading to the Archaea and Eukaryotes.

### Automatic OGD Detection

Our automatic analysis compares the signal from individual gene families to the combined phylogenetic signal from all 40 single copy genes. We test each individual branch of the combined tree (internal and external) to see if the gene family supports its placement in the overall phylogeny. For each branch we expect one of three results: 1) That the gene family supports the same placement of the branch as in the combined tree; 2) That the gene family will not have enough phylogenetic signal to determine a good placement for the branch; 3) That the gene family supports with high confidence an alternative placement of the branch. This final type of result indicates incongruence within the gene family that may have been caused by horizontal transfer. The theory behind this approach is that phylogenetic signal is cumulative as opposed to homoplastic noise which is dispersive [28], therefore strong disagreement between the combined phylogenetic signal of all the gene families and that of any one gene may be representative of a homoplastic event like HGT [29].

An individual tree was created for every possible alternative position of each (internal and external) branch of the unrooted combined phylogeny (see figure 1 for an example). The unrooted 191 taxon combined tree has 379 (internal and external) branches; so each of these branches has up to 377 alternative positions, depending on the size of the clade it defines (including its original position). The total number of trees tested with each of the 40 gene families was 140,005.

For each individual gene family, the sequences from all three domains of life were aligned using the default settings in muscle [37]. This alignment was then used to assess each of the possible alternative trees. This was done using an in-house script that generated a file containing an unrooted tree for every alternative position of the branch on the combined tree (including its original position). This file was used as input for Tree-Puzzle[42] along with the alignment from the gene family using the default settings except specifying the JTT model of evolution. All the alternative phylogenies were assessed and the expected likelihood weight (ELW) [31] calculated by puzzle was used to assess the confidence of each tree. This tested the placement of the branch in each alternative position. In likelihood weighting each sampled tree is weighted by the likelihood that it accords with the evidence (the alignment). Trees for which the alignment is unlikely are given less weight. The sum of the weights calculated for all the trees tested equals 1. This allows the quantification of the weight of evidence supporting each of the un-rejected trees and their ranking according to their support of the evidence in the alignment [31]. For example: A tree with a value of 0.65 represents a single un-rejected tree that contributes 65% of the total weighted evidence in support of the alignment from all the un-rejected trees. This approach has been used previously [30] to infer species compositions from metagenomics samples where the lack of sequenced representatives of the sampled organisms can cause uncertainty in their phylogenetic placement. This is analogous to the situation where there may be no sequenced representative of the donor organism of a horizontally transferred gene.

Next, the path length distance [32] (representing substitutions per site) across the tree from the best placement of the branch to its placement on the combined signal tree was calculated (if they differed) using an in-house script. This was used to try to distinguish horizontal transfer from phylogenetic uncertainty (Figure 4).

It has been previously noted that for various reasons (including missing sampled representative of the donor organism or settings that are too strict) phylogenetic methods of detecting HGTs can underestimate the number of these events that have occurred in a dataset [43]. To explore this possibility and to determine optimum settings for use in our analysis we carried out a parameter exploration of all combinations of ELW values (from 0.55 to 1.0 in steps of 0.5) and path length distances (from 0.1 to 1.0 in steps of 0.1) to see the effect of their use as cut-offs on the number of OGDs identified. For example: only allowing HGTs that have an ELW of 0.2 or above and have occurred between branches of the tree a minimum path length distance of 0.6 identified 61 putative OGDs (Figure 2 and S6). This resulted in the identification of a range of settings where the number OGDs plateaued (Figure 2). We chose one of the most conservative of these settings, an expected likelihood weight (ELW) of 0.65 and a minimum the path length distance across the tree from the best placement of the branch to its placement on the combined tree of 0.40 (representing substitutions per site), to identify putative HGTs for further analysis (Figure 2).

The consequence of these settings was if for any branch there was no placement with an ELW of 0.65 or greater, the gene family was considered not to have enough phylogenetic information to determine the correct placement of the branch. Also, if the path length distance from the best placement of the branch to its original position on the combined tree was less than 0.40 (representing substitutions per site) it was considered too close to its original position to exclude phylogenetic uncertainty as a cause. However, if both these prerequisites were met, the placement was considered a putative horizontal transfer in this gene family.

For all putative OGDs identified a series of rules were applied to filter out events that were unlikely to be real. Firstly, if for a single gene family a “double” transfer was hypothesised, where two branches where both each other's donor and recipient, both were disregarded as the direction of transfer could not be identified. Secondly, transfers that originated in an ancestral branch of the recipient were also disregarded, even though this could be evidence of a transfer from an organism similar to the ancestor, but for which we do not have a sequenced genome.

This process identified 68 putative orthologous gene displacement events in the 40 gene families analysed (Figure 3). A previous manual analysis of 31 of these gene families had identified 7 transfer events [14], all of which were contained within the 38 OGDs we identified from the same gene families using the same species set. The large increase in the number of OGD events detected is not surprising as the previous analysis [14] was based building trees with concatenated alignments of random subsets of the 31 genes. They identified by eye situations where the relationships in the phylogeny changed after certain gene families were removed from the concatenated alignment. The species(s) that changed position were hypothesised to have undergone a HGT event in the gene family that was removed. This approach depends on the jack-knifing approach having selected the correct set of genes to build the tree to identify the difference in phylogeny with confidence. This is likely to vastly underestimate the number of HGTs detected.

Furthermore we also identified a further 30 novel OGDs from the additional 9 gene families analysed here (Table 1).

### Genome size

The genome size of putative ancestors was calculated for every internal branch of the tree by taking the average genome size (in Mb) of the organisms in the clade defined by the internal branch. The information on genome size was taken from the NCBI microbial genome website (http://www.ncbi.nlm.nih.gov/genomes/lproks.cgi). A Wilcoxon rank sum test with continuity correction was carried out using the statistics package “R” (http://www.r-project.org/), to determine if the null hypothesis that the genome size of the acceptors and donors were the same, could be rejected. We found that the null hypothesis was rejected and that acceptors were larger than donors at a significance level of 0.02 (Figure 4B). This approach could be affected by the sampling of the genomes in each clade, but serves an indication of possible trends in the data.

### Evolutionary distance

Using the tree in Figure 3, we calculated the path length distances across the tree the donor and acceptor of each detected HGT event (representing evolutionary distance). Next, the path length between all possible donors and acceptors were calculated. This amounted to 103,210 comparisons based on the 191 taxa tree in figure 3. We tested to see if the distribution of evolutionary distances between the observed donors and acceptors were significantly different to the expected, given the shape and structure of the tree in figure 3. Using the Kolmogorov-Smirnov test we found that the distance between the donors and acceptors of the observed OGDs were significantly shorter than expected with a p value of 0.001 (Figure 4C).

### Rate of HGT events

n order to make a statement about the rate of OGDs, it was necessary to see if the branch lengths in figure 3 approximately represented time. Horizontal transfers are unique markers for dating parts of a tree. The existence of a transfer indicates that two distinct parts of the tree (the donor and acceptor) existed at the same time and in the same place (or in the presence of an intermediary). If the branch lengths are clock-like across the entire tree, the path length distances from the donor and acceptor to their last common ancestor should be the same (taking into account that the HGT event could have taken place at any stage along the donor or acceptor branch). We found that this held for 53 of the 68 OGDs calculated indicating that these branches were good representations of time since they last shared a common ancestor (Table S1). To demonstrate this concept graphically we joined the donor and acceptor for each transfer with an ELW greater 0.9 with lines perpendicular to time (Figure S5) and found with the exception of two branches (leading to the Crenarchaea and Leptospira/Spirochaetaceae clades), the branch lengths were a good representation of time.

Next, for each branch of the tree we calculated the total of the branch lengths in the clade it defined (if it was an internal branch, otherwise we used the branch length of the leaf). We then plotted these calculations against the number of OGDs accepted into each clade (the grey plot in Figure 4d). However, as the clades are nested within each other on the tree, we chose 15 clades representing independent major groups of organisms for statistical tests (plotted in black in Figure 4d). These groups were Clostridia, Mollicutes, Bacilli, Bacteroides, Chlamydia, Planctomycetes, Leptospira, Spirochaeta, Actinobacteria, Cyanobacteria, Acidobacteria, Proteobacteria, Crenarcheaota, Euryarchaeota, and Eukaryotes. We found that there was a significant correlation between the total evolutionary time/opportunity and the number of HGTs that occurred in these independent groups (p = 0.005, using the statistical package “R” (http://www.r-project.org/)), and that this data was linear (p<0.005 using the linearity test from the tseries package in R, and p>0.5 using the runs test from the car package [44] in R (Figures S6, S7 and S8).

### GC Content analysis

The average GC contents of the 191 genomes used in the analysis were retrieved from the NCBI database (www.ncbi.nlm.nih.gov/genomes). Using the tree in figure 3, we estimated the average GC content at each internal branch by taking a simple average of the GC contents of the species contained in the clade defined by the internal branch. The GC content was checked for significant correlations both between the donors and acceptors of each OGD identified and between all donors and all acceptors (Figure S1). This analysis is likely to be affected by sampling bias, but still has the potential to reveal overall trends.

### Habitat analysis

Information on the habitat distribution of the 191 species used in the analysis was retrieved from type culture collections and summarused into 6 categories of habitat (Aquatic, Extreme, Foodstuff, Internal, Agricultural runoff and Terrestrial). We recorded the number of times any species was recoreded in any of these broad categories of habitats. This gave us an indication of the specialisation of each organism in the analysis. Next for each internal branch we summed the totals for the 6 categories of all the organisms in the clade it defined. This was used as the estimate of the habitat range of the hypothetical ancestor at the internal branch.

These counts were converted to proportions of the total number of observations for each species and internal branch on the tree. This allowed us to calculate the 6D (euclidean) “habitat distance” between any two branches on the tree in figure 1. We used this information to compare the habitat of the donors and acceptors of each of the OGDs identified. This was compared to the evolutionary distance from the tree in figure 1 between the donors and acceptors of each OGD (Figure S2).

## Supporting Information

Figure S1 **The %GC Content of Donors and Acceptors.** There was no significant difference in GC content between the donors and acceptors of the 68 OGDs detected.
(PDF)

Figure S2 **The Comparison of predicted habitats of donors and acceptors.** The predicted habitats were calculated for the donors and acceptors of each of the 68 OGDs found using their habitat information as available from cultivated strains in culture collections. The information was summarised into 6 categories (Aquatic, Extreme, Foodstuff, Internal, Agricultural runoff and Terrestrial) and the number of times each species was identified as being present in each habitat category was recorded. The same numbers were calculated for each internal branch of the tree in figure 3 by summing the number of observations for all the species contained in the clade defined by the internal branch. These numbers were converted to proportions of the total number of observations for the species/internal branch. We then calculated the 6D distance of the habitat distribution between the donor and acceptor of each OGD identified. This was compared to the evolutionary distance between the donor and acceptor as calculated from the tree in figure 3. We found no correlation between the similarity of the habitats between donors and acceptors and their evolutionary distance.
(PDF)

Figure S3 **The number of OGDs identified versus the number of protein interactions predicted for each of the genes.** The number of interactions was calculated for each of the 40 gene families using STRING 7.0 [33] using a cut-off of 0.7. The negative correlation between the number of interactions and the number of OGDs is significant with a P-value of 0.0008 using Pearson's correlation coefficient.
(PDF)

Figure S4 **OGDs mapped onto the assembly maps of the ribosomal subunits.** The assembly maps of A) the small-subunit and B) the large subunit of the ribosome. For clarity, only the strong interactions are shown in B). In both A and B the numbers represent the protein names of each sub-unit. The proteins are coloured according to the number of OGDs found. Those proteins in grey were not considered in this analysis because of being in multi-copy or not being universal. Proteins in Blue, Yellow, Orange and Red had 0, 1, 2 and 3 OGDs accordingly.
(PDF)

Figure S5 **The most highly supported OGDs plotted as time-points onto the tree.** The OGDs with greater than 0.9 ELW mapped onto the tree constructed from the combined phylogenetic information from all 40 genes used in the study. In general the OGDs mapped perfectly onto the tree without adjustment, except for two branches which needed to be extended (marked by a dotted line).
(PDF)

Figure S6 **Results of parameter permutation.** To identify the best setting for the analysis permutation of the variables was performed and the results analysed. By default we used a branch length distance of 0.4 (substitutions per site) and an ELW score of 0.65 as cut-offs to identify putative OGDs (outlined in black in the figure). We calculated the number of OGDs found using 100 different combinations of both these values, each of which was tested for a linear rate of occurrence. The numbers in the table represent the number of OGDs found and the colour of the box represents the statistical support for the fit of the data to a linear model (as calculated in R). The data was also shown to be linear (Figure S7 and S8).
(PDF)

Figure S7 **Results of linearity test.** The numbers in the table represent the number of OGDs found and the colour of the box represents the statistical support for linearity in the data as calculated with the linearity test (as implemented in the car [44] package in R).
(PDF)

Figure S8 **Results of runs test for linearity.** The numbers in the table represent the number of OGDs found and the colour of the box represents the statistical support for linearity in the data as calculated with the runs test (as implemented in the tseries package in R).
(PDF)

Table S1 **Details of the 68 OGD events detected as part of this study.** For each OGD event detected the gene family ID (COGID) and annotated function is displayed along with the estimated donor and acceptor and the expected likelihood weight (ELW) and path length distance calculated. Also displayed are the path length distance from the last common ancestor (LCA) of the donor and acceptor for each OGD to the midpoint of the donor and acceptor branch, along with the length of the donor and acceptor branch. Finally “Is Overlapped?” indicates whether the branch lengths to the donor and acceptor from their LCA overlap, indicating the branches involved are representative of the amount of time passed since they last shared a common ancestor.
(PDF)
