# Supplementary figures and images for: Universally Distributed Single-Copy Genes Indicate a Constant Rate of Horizontal Transfer
Source: PLoS One. 2011 Aug 5;6(8):e22099. doi: 10.1371/journal.pone.0022099 (PMC3151239; doi:10.1371/journal.pone.0022099)

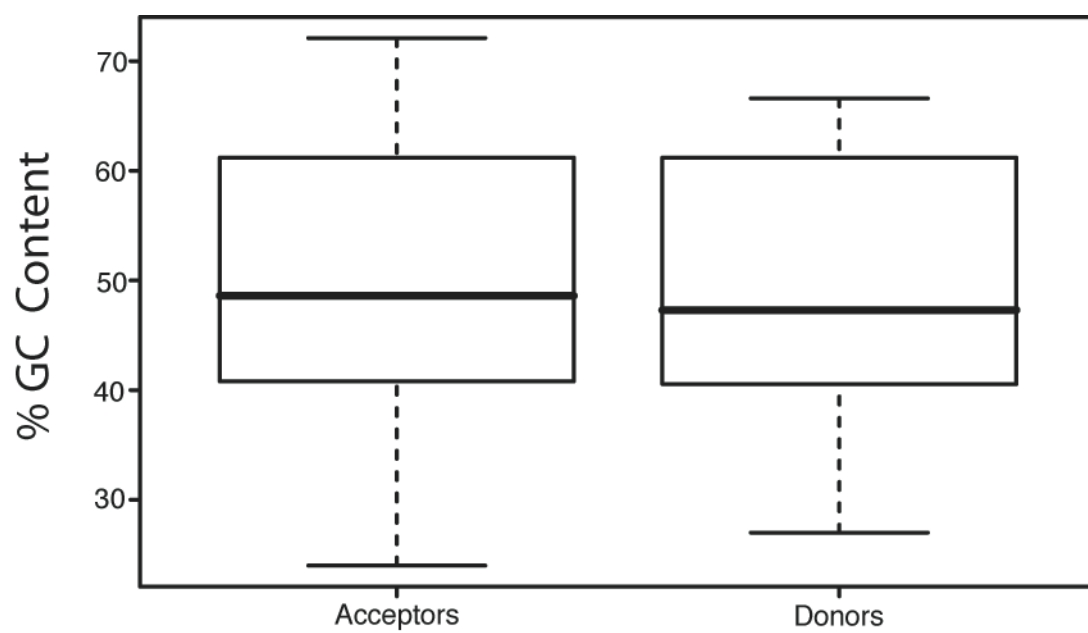

Supplement: Figure S1 — The %GC Content of Donors and Acceptors. There was no significant difference in GC content between the donors and acceptors of the 68 OGDs detected. (PDF) [file pone.0022099.s001.pdf]

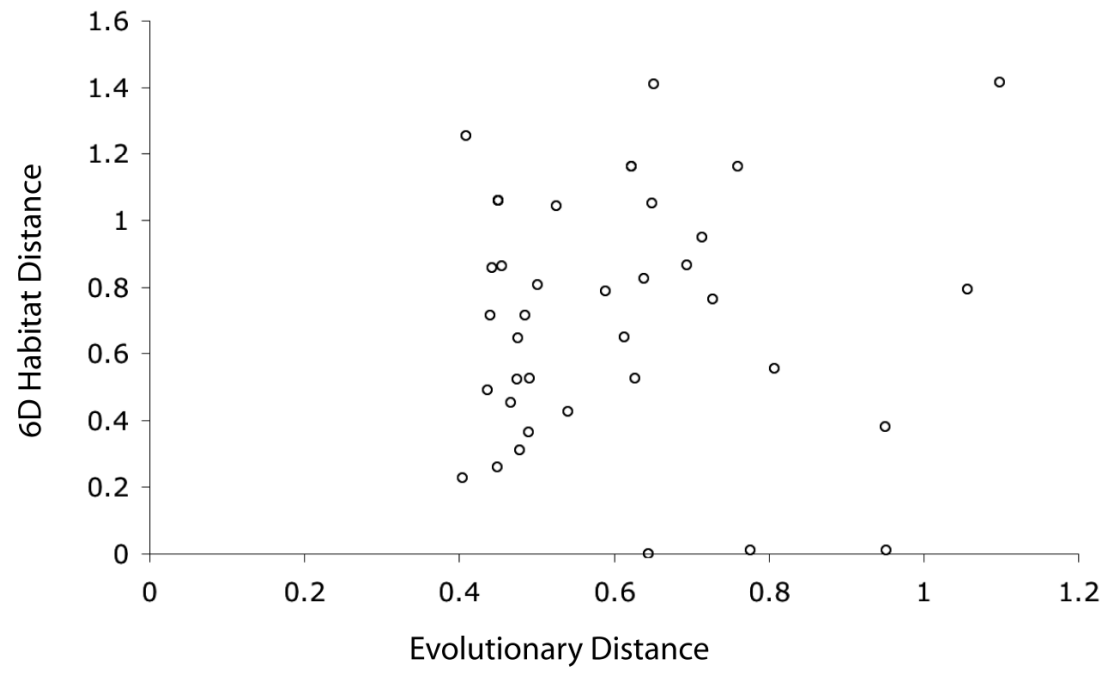

Supplement: Figure S2 — The Comparison of predicted habitats of donors and acceptors. The predicted habitats were calculated for the donors and acceptors of each of the 68 OGDs found using their habitat information as available from cultivated strains in culture collections. The information was summarised into 6 categories (Aquatic, Extreme, Foodstuff, Internal, Agricultural runoff and Terrestrial) and the number of times each species was identified as being present in each habitat category was recorded. The same numbers were calculated for each internal branch of the tree in figure 3 by summing the number of observations for all the species contained in the clade defined by the internal branch. These numbers were converted to proportions of the total number of observations for the species/internal branch. We then calculated the 6D distance of the habitat distribution between the donor and acceptor of each OGD identified. This was compared to the evolutionary distance between the donor and acceptor as calculated from the tree in figure 3. We found no correlation between the similarity of the habitats between donors and acceptors and their evolutionary distance. (PDF) [file pone.0022099.s002.pdf]

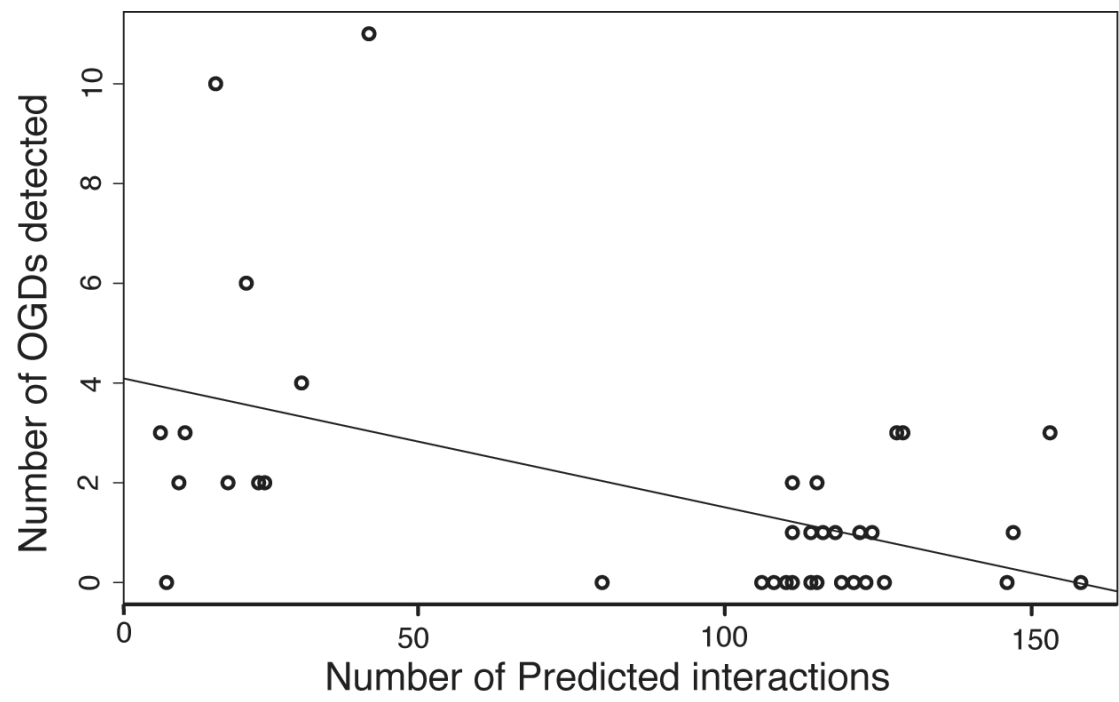

Supplement: Figure S3 — The number of OGDs identified versus the number of protein interactions predicted for each of the genes. The number of interactions was calculated for each of the 40 gene families using STRING 7.0 [33] using a cut-off of 0.7. The negative correlation between the number of interactions and the number of OGDs is significant with a P-value of 0.0008 using Pearson's correlation coefficient. (PDF) [file pone.0022099.s003.pdf]

**A**

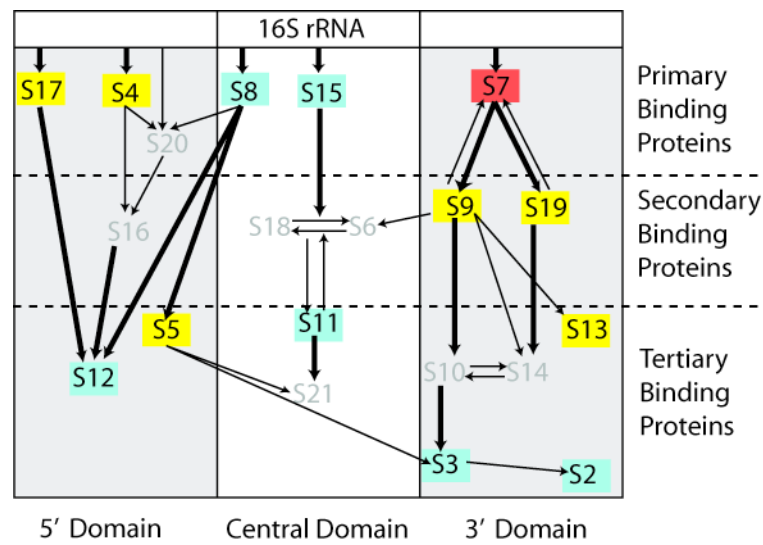

**B**

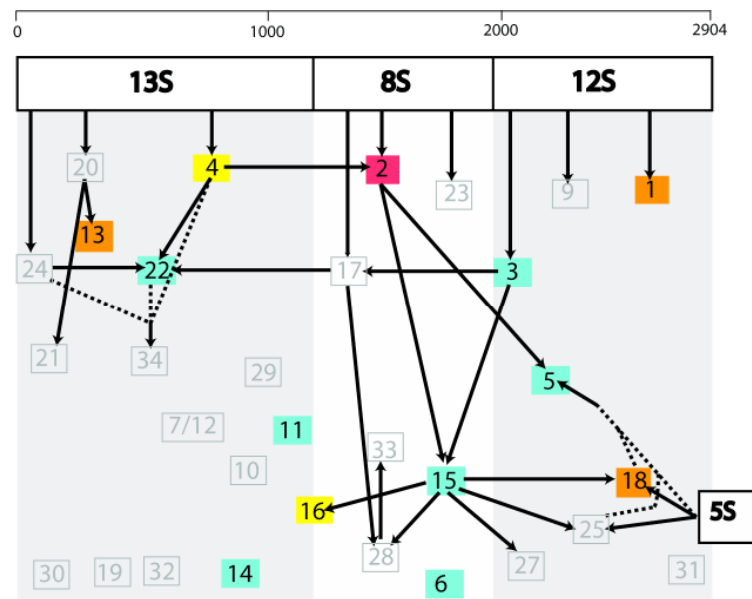

Supplement: Figure S4 — OGDs mapped onto the assembly maps of the ribosomal subunits. The assembly maps of A) the small-subunit and B) the large subunit of the ribosome. For clarity, only the strong interactions are shown in B). In both A and B the numbers represent the protein names of each sub-unit. The proteins are coloured according to the number of OGDs found. Those proteins in grey were not considered in this analysis because of being in multi-copy or not being universal. Proteins in Blue, Yellow, Orange and Red had 0, 1, 2 and 3 OGDs accordingly. (PDF) [file pone.0022099.s004.pdf]

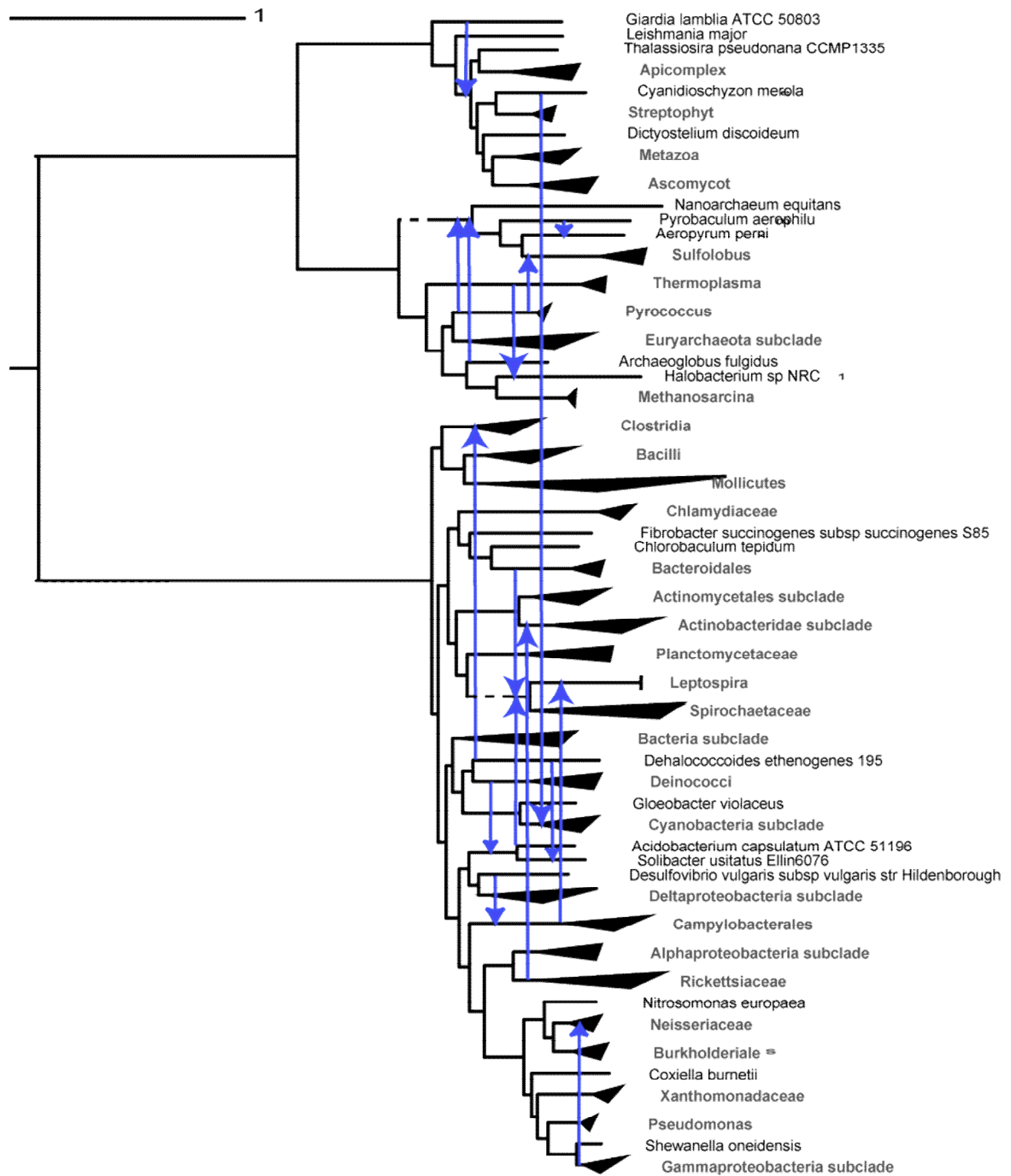

Supplement: Figure S5 — The most highly supported OGDs plotted as time-points onto the tree. The OGDs with greater than 0.9 ELW mapped onto the tree constructed from the combined phylogenetic information from all 40 genes used in the study. In general the OGDs mapped perfectly onto the tree without adjustment, except for two branches which needed to be extended (marked by a dotted line). (PDF) [file pone.0022099.s005.pdf]

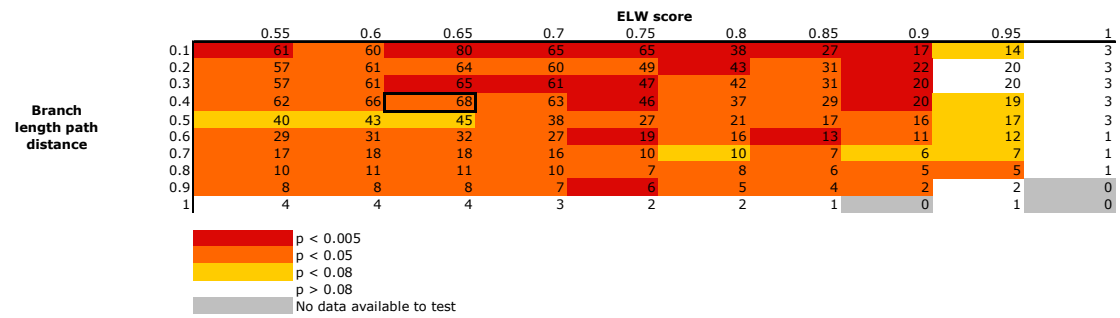

Supplement: Figure S6 — Results of parameter permutation. To identify the best setting for the analysis permutation of the variables was performed and the results analysed. By default we used a branch length distance of 0.4 (substitutions per site) and an ELW score of 0.65 as cut-offs to identify putative OGDs (outlined in black in the figure). We calculated the number of OGDs found using 100 different combinations of both these values, each of which was tested for a linear rate of occurrence. The numbers in the table represent the number of OGDs found and the colour of the box represents the statistical support for the fit of the data to a linear model (as calculated in R). The data was also shown to be linear (Figure S7 and S8). (PDF) [file pone.0022099.s006.pdf]

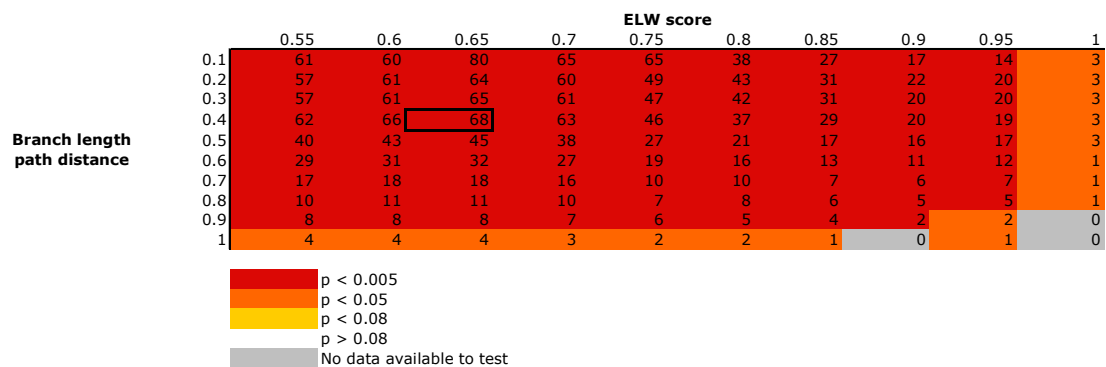

Supplement: Figure S7 — Results of linearity test. The numbers in the table represent the number of OGDs found and the colour of the box represents the statistical support for linearity in the data as calculated with the linearity test (as implemented in the car [44] package in R). (PDF) [file pone.0022099.s007.pdf]

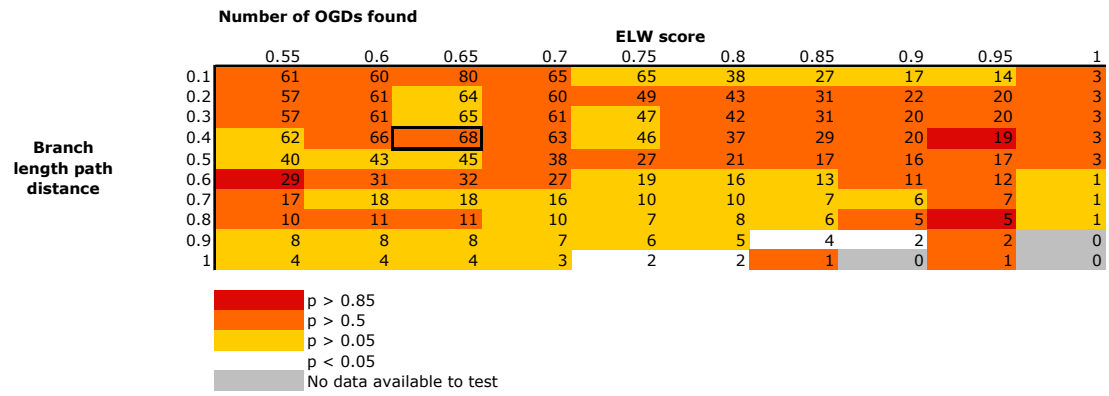

Supplement: Figure S8 — Results of runs test for linearity. The numbers in the table represent the number of OGDs found and the colour of the box represents the statistical support for linearity in the data as calculated with the runs test (as implemented in the tseries package in R). (PDF) [file pone.0022099.s008.pdf]
