# Supplementary material for: Universally Distributed Single-Copy Genes Indicate a Constant Rate of Horizontal Transfer
Source: PLoS One. 2011 Aug 5;6(8):e22099. doi: 10.1371/journal.pone.0022099 (PMC3151239; doi:10.1371/journal.pone.0022099)
Supplement: Table S1 — Details of the 68 OGD events detected as part of this study. For each OGD event detected the gene family ID (COGID) and annotated function is displayed along with the estimated donor and acceptor and the expected likelihood weight (ELW) and path length distance calculated. Also displayed are the path length distance from the last common ancestor (LCA) of the donor and acceptor for each OGD to the midpoint of the donor and acceptor branch, along with the length of the donor and acceptor branch. Finally “Is Overlapped?” indicates whether the branch lengths to the donor and acceptor from their LCA overlap, indicating the branches involved are representative of the amount of time passed since they last shared a common ancestor. (PDF) [file pone.0022099.s009.pdf]

COGID	Annotated Function	Doner	Acceptor	ELW	Path length distance	Midpoint donor branch to ancestor	Midpoint acceptor branch to ancestor	Donor branch length	Acceptor branch length	Is Over-lapped?
**COG0012**	GTP Binding Protein (GTPase, probable translation factor)	*Aeropyrum pernix*	*Euryarchaeota subclade*	0.7174	0.474323	0.491237	0.334959	0.04731	0.03407	FALSE
**COG0012**	GTP Binding Protein (GTPase, probable translation factor)	*Bacteria subclade*	*Alphaproteobacteria*	0.7942	0.714165	0.377645	0.147717	0.18375	0.0315	FALSE
**COG0012**	GTP Binding Protein (GTPase, probable translation factor)	*Geobacter sulfurreducens*	*Candidatus Solibacter usitatus Ellin6076*	0.8592	0.421942	0.110782	0.021069	0.22966	0.10257	TRUE
**COG0012**	GTP Binding Protein (GTPase, probable translation factor)	*Deinococci*	*Acidobacterium capsulatum ATCC 51196*	0.9586	0.832381	0.131588	0.320149	0.26993	0.14542	TRUE
**COG0012**	GTP Binding Protein (GTPase, probable translation factor)	*Proteobacteria subclade*	*Campylobacter jejuni*	0.9707	0.525971	0.110899	0.072029	0.1916	0.20695	TRUE
**COG0012**	GTP Binding Protein (GTPase, probable translation factor)	*Bacteria subclade*	*Rickettsiales*	0.9911	0.807496	0.377645	0.210728	0.18375	0.06182	TRUE
**COG0016**	Phenylalanyl-tRNA synthetase alpha subunit	*Pyrococcus furiosus*	*Borrelia burgdorferi*	0.722	3.406985	2.901763	0.222732	0.1811	0.10139	FALSE
**COG0016**	Phenylalanyl-tRNA synthetase alpha subunit	*Acidobacterium capsulatum ATCC 51196*	*Spirochaetaceae*	0.9492	0.642963	0.350726	0.137025	0.14542	0.03624	FALSE
**COG0016**	Phenylalanyl-tRNA synthetase alpha subunit	*Pyrococcus furiosus*	*Thermoprotei*	0.9814	0.489491	0.4478	0.216634	0.1811	0.07721	TRUE
**COG0018**	Arginyl-tRNA synthetase	*Gammaproteobacteria subclade*	*Fibrobacter succinogenes subsp succinogenes S85*	0.688	0.979893	0.413132	0.241279	0.16592	0.18601	TRUE
**COG0018**	Arginyl-tRNA synthetase	*Streptophyta*	*Gloeobacter violaceus*	0.8195	3.532069	3.086301	0.131678	0.19159	0.1225	FALSE
**COG0018**	Arginyl-tRNA synthetase	*Campylobacter jejuni*	*Thermotoga maritima*	0.867	0.408469	0.117135	0.08061	0.20695	0.03848	TRUE
**COG0018**	Arginyl-tRNA synthetase	*Desulfovibrio vulgaris str Hildenborough*	*Betaproteobacteria*	0.876	0.436147	0.159602	0.135042	0.11245	0.08456	TRUE
**COG0018**	Arginyl-tRNA synthetase	*Borrelia burgdorferi*	*Bacteroides thetaiotaomicron*	0.8867	0.613513	0.174864	0.206629	0.10139	0.17347	TRUE

**COG0018**	Arginyl-tRNA synthetase	*Vibrionaceae*	*Chromobacterium violaceum*	0.9213	0.404419	0.309856	0.210579	0.01746	0.03565	FALSE
**COG0018**	Arginyl-tRNA synthetase	*Bacteroides thetaiotaomicron*	*Spirochaetaceae*	0.926	0.47588	0.206629	0.102381	0.17347	0.03624	TRUE
**COG0018**	Arginyl-tRNA synthetase	*Deinococcus radiodurans*	*Candidatus Solibacter usitatus Ellin6076*	0.9306	0.438323	0.13158	0.114999	0.12388	0.10257	TRUE
**COG0018**	Arginyl-tRNA synthetase	*Campylobacter jejuni*	*Leptospira interrogans*	0.9547	0.775583	0.147712	0.209509	0.20695	0.23786	TRUE
**COG0018**	Arginyl-tRNA synthetase	*Streptophyta*	*Cyanobacteria subclade*	0.9571	3.692804	3.086301	0.376683	0.19159	0.03823	FALSE
**COG0049**	Ribosomal protein S7	*Methanothermobacter thermautotrophicus str Delta H*	*Thermoprotei*	0.7242	0.622339	0.585098	0.216634	0.17665	0.07721	FALSE
**COG0049**	Ribosomal protein S7	*Methanothermobacter thermautotrophicus str Delta H*	*Pyrobaculum aerophilum*	0.8263	0.759644	0.585098	0.371069	0.17665	0.06008	TRUE
**COG0049**	Ribosomal protein S7	*Thermoplasma acidophilum*	*Euryarchaeota subclade*	0.8638	0.450882	0.059171	0.127319	0.33075	0.05197	TRUE
**COG0081**	Ribosomal protein L1	*Bacteria subclade*	*Pirellula sp*	0.6631	0.554028	0.114269	0.102389	0.25991	0.1203	TRUE
**COG0081**	Ribosomal protein L1	*Thermoplasma acidophilum*	*Euryarchaeota subclade*	0.7663	0.450882	0.059171	0.127319	0.33075	0.05197	TRUE
**COG0088**	Ribosomal protein L4	*Methanocaldococcus jannaschii*	*Pyrobaculum aerophilum*	0.8228	0.627674	0.510648	0.371069	0.11913	0.06008	TRUE
**COG0090**	Ribosomal protein L2	*Deinococci*	*Bacteria subclade*	0.6915	0.466903	0.175389	0.000004	0.26993	0.02158	TRUE
**COG0090**	Ribosomal protein L2	*Deinococci*	*Gloeobacter violaceus*	0.7049	0.436147	0.065374	0.021663	0.26993	0.1225	TRUE
**COG0090**	Ribosomal protein L2	*Pyrococcus furiosus*	*Eukaryota*	0.7525	0.9721	0.4478	0.371062	0.1811	0.40539	TRUE
**COG0098**	Ribosomal protein S5	*Gloeobacter violaceus*	*Aquifex aeolicus*	0.764	0.449752	0.056924	0.126618	0.1225	0.17091	TRUE
**COG0099**	Ribosomal protein S13	*Methanothermobacter thermautotrophicus str Delta H*	*Thermoprotei*	0.7598	0.622339	0.585098	0.216634	0.17665	0.07721	FALSE
**COG0102**	Ribosomal protein L13	*Bacteria subclade*	*Ureaplasma parvum*	0.7564	1.098876	0.39087	0.428377	0.18375	0.09588	TRUE
**COG0102**	Ribosomal protein L13	*Proteobacteria subclade*	*Betaproteobacteria*	0.8928	0.466594	0.110899	0.135042	0.1916	0.08456	TRUE
**COG0103**	Ribosomal protein S9	*Thermoplasma acidophilum*	*Euryarchaeota subclade*	0.8642	0.450882	0.059171	0.127319	0.33075	0.05197	TRUE

**COG0124**	Histidyl-tRNA synthetase	*Betaproteobacteria*	*Gloeobacter violaceus*	0.6681	0.44038	0.180149	0.087877	0.08456	0.1225	TRUE
**COG0124**	Histidyl-tRNA synthetase	*Spirochaetales*	*Campylobacter jejuni*	0.6871	0.44216	0.090876	0.147712	0.02307	0.20695	TRUE
**COG0124**	Histidyl-tRNA synthetase	*Bacteria subclade*	*Leptospira interrogans*	0.7001	0.744065	0.114269	0.174865	0.25991	0.23786	TRUE
**COG0124**	Histidyl-tRNA synthetase	*Xanthomonas*	*Bacteria subclade*	0.7159	1.056467	0.407306	0.503056	0.12075	0.06006	TRUE
**COG0124**	Histidyl-tRNA synthetase	*Cyanobacteria subclade*	*Alphaproteobacteria*	0.7245	0.485046	0.332881	0.11714	0.03823	0.0315	FALSE
**COG0124**	Histidyl-tRNA synthetase	*Spirochaetales*	*Caulobacter vibrioides*	0.7716	0.478157	0.090876	0.33438	0.02307	0.05628	FALSE
**COG0124**	Histidyl-tRNA synthetase	*Wolinella succinogenes*	*Spirochaetales*	0.8145	0.694638	0.56162	0.090876	0.04552	0.02307	FALSE
**COG0124**	Histidyl-tRNA synthetase	*Deinococcus radiodurans*	*Streptomyces*	0.8443	0.649007	0.162157	0.358879	0.12388	0.03054	FALSE
**COG0124**	Histidyl-tRNA synthetase	*Bacteroides thetaiotaomicron*	*Cyanobacteria subclade*	0.8575	0.870574	0.241273	0.363459	0.17347	0.11882	TRUE
**COG0124**	Histidyl-tRNA synthetase	*Thermoplasma acidophilum*	*Archaeoglobus fulgidus*	0.982	0.938844	0.059171	0.35927	0.33075	0.30798	TRUE
**COG0124**	Histidyl-tRNA synthetase	*Eukaryota subclade*	*Eukaryota subclade*	0.9976	0.410971	0.287725	0.389007	0.2774	0.03227	TRUE
**COG0172**	Seryl-tRNA synthetase	*Euryarchaeota subclade*	*Pyrococcus furiosus*	0.836	0.503046	0.138025	0.078771	0.1733	0.1811	TRUE
**COG0172**	Seryl-tRNA synthetase	*Euryarchaeota subclade*	*Thermoprotei*	0.998	0.540946	0.507055	0.216634	0.1733	0.07721	FALSE
**COG0185**	Ribosomal protein S19	*Tropheryma whipplei str Twist*	*Streptomyces coelicolor*	0.6688	0.512534	0.27829	0.195104	0.22361	0.08353	TRUE
**COG0186**	Ribosomal protein S17	*Bacteria subclade*	*Actinomycetales subclade*	0.7808	0.521565	0.114269	0.056225	0.25991	0.134	TRUE
**COG0197**	Ribosomal protein L16/L10E	*Arabidopsis thaliana*	*Eukaryota subclade*	0.7849	0.629788	0.561254	0.287725	0.07884	0.2774	TRUE
**COG0202**	DNA-directed RNA polymerase, alpha subunit/40 kD subunit	*Pyrococcus furiosus*	*Thermoplasma acidophilum*	0.7015	0.624703	0.17201	0.059171	0.1811	0.33075	TRUE
**COG0202**	DNA-directed RNA polymerase, alpha subunit/40 kD subunit	*Eukaryota*	*Methanocaldococcus jannaschii*	0.7035	0.972977	0.371062	0.510648	0.40539	0.11913	TRUE
**COG0202**	DNA-directed RNA polymerase, alpha subunit/40 kD subunit	*Methanocaldococcus jannaschii*	*Thermoprotei*	0.8459	0.490369	0.510648	0.216634	0.11913	0.07721	FALSE
**COG0215**	Cysteinyl-tRNA synthetase	*Chlamydia*	*Methanosarcina*	0.6822	3.439583	0.113724	2.961018	0.30084	0.064	FALSE
**COG0215**	Cysteinyl-tRNA	*Leptospira*	*Candidatus Solibacter*	0.82	0.669067	0.209509	0.145576	0.23786	0.10257	TRUE

	synthetase	*interrogans*	*usitatus Ellin6076*							
**COG0256**	Ribosomal protein L18	*Treponema*	*Campylobacter jejuni*	0.66	0.952114	0.412289	0.147712	0.21161	0.20695	TRUE
**COG0256**	Ribosomal protein L18	*Campylobacter jejuni*	*Borrelia burgdorferi*	0.6809	0.639112	0.147712	0.209508	0.20695	0.10139	TRUE
**COG0495**	Leucyl-tRNA synthetase	*Geobacter sulfurreducens*	*Aquifex aeolicus*	0.7118	0.728146	0.204712	0.157571	0.22966	0.17091	TRUE
**COG0495**	Leucyl-tRNA synthetase	*Deinococci*	*Clostridium*	0.9332	0.547427	0.175389	0.043178	0.26993	0.05893	TRUE
**COG0522**	Ribosomal protein S4 and related proteins	*Eukaryota*	*Pyrococcus*	0.7199	0.768651	0.371062	0.403112	0.40539	0.02234	TRUE
**COG0525**	Valyl-tRNA synthetase	*Pyrococcus furiosus*	*Methanothermobacter thermautotrophicus str Delta H*	0.6887	0.495051	0.022349	0.159646	0.1811	0.17665	TRUE
**COG0525**	Valyl-tRNA synthetase	*Rickettsia*	*Bifidobacterium longum*	0.9905	0.799228	0.334383	0.358873	0.06028	0.07214	TRUE
**COG0525**	Valyl-tRNA synthetase	*Pyrococcus furiosus*	*Sulfolobus solfataricus*	0.9905	0.950104	0.4478	0.585857	0.1811	0.1686	TRUE
**COG0525**	Valyl-tRNA synthetase	*Thermoprotei subclade*	*Sulfolobus*	0.997	0.589089	0.060088	0.154716	0.27757	0.21689	TRUE
**COG0533**	Metal-dependent proteases with possible chaperone activity	*Bacteria subclade*	*Mycoplasma mycoides subsp mycoides SC*	0.6895	0.644746	0.124416	0.22956	0.22807	0.0627	TRUE
**COG0533**	Metal-dependent proteases with possible chaperone activity	*Campylobacter jejuni*	*Fusobacterium nucleatum subsp nucleatum*	0.8295	0.651876	0.117135	0.157577	0.20695	0.20492	TRUE
**COG0533**	Metal-dependent proteases with possible chaperone activity	*Bacteria subclade*	*Clostridium*	0.8437	0.454593	0.124416	0.043178	0.22807	0.05893	TRUE
**COG0541**	Signal recognition particle GTPase	*Thermoplasma acidophilum*	*Euryarchaeota subclade*	0.6982	0.450882	0.059171	0.127319	0.33075	0.05197	TRUE
**COG0541**	Signal recognition particle GTPase	*Pyrococcus furiosus*	*Thermoplasma*	0.8294	0.501314	0.022349	0.15965	0.1811	0.18291	TRUE
